# On the Dual Role of Carcinoembryonic Antigen-Related Cell Adhesion Molecule 1 (CEACAM1) in Human Malignancies

**DOI:** 10.1155/2018/7169081

**Published:** 2018-10-14

**Authors:** Andreea Calinescu, Gabriela Turcu, Roxana I. Nedelcu, Alice Brinzea, Anastasia Hodorogea, Mihaela Antohe, Carmen Diaconu, Coralia Bleotu, Daniel Pirici, Lucia B. Jilaveanu, Daniela A. Ion, Ioana A. Badarau

**Affiliations:** ^1^Carol Davila University of Medicine and Pharmacy, 050474 Bucharest, Romania; ^2^Dermatology 1 Department, Colentina Clinical Hospital, 020125 Bucharest, Romania; ^3^Derma 360° Clinic, 011273 Bucharest, Romania; ^4^National Institute for Infectious Diseases Prof. Dr. Matei Balș, 021105 Bucharest, Romania; ^5^Stefan Nicolau Institute of Virology, 030304 Bucharest, Romania; ^6^University of Medicine and Pharmacy of Craiova, 200349 Craiova, Romania; ^7^Department of Medicine, Section of Medical Oncology, Yale University School of Medicine, New Haven, CT 208028, USA

## Abstract

Carcinoembryonic antigen-related cell adhesion molecule 1 (CEACAM1) is a glycoprotein belonging to the carcinoembryonic antigen (CEA) family that is expressed on a wide variety of cells and holds a complex role in inflammation through its alternate splicing and generation of various isoforms, mediating intricate mechanisms of modulation and dysregulation. Initially regarded as a tumor suppressor as its expression shows considerable downregulation within the epithelia in the early phases of many solid cancers, CEACAM1 has been linked lately to the progression of malignancy and metastatic spread as various papers point to its role in tumor progression, angiogenesis, and invasion. We reviewed the literature and discussed the various expression patterns of CEACAM1 in different types of tumors, describing its structure and general biologic functions and emphasizing the most significant findings that link this molecule to poor prognosis. The importance of understanding the role of CEACAM1 in cell transformation stands not only in this adhesion molecule's value as a prognostic factor but also in its promising premise as a potential new molecular target that could be exploited as a specific cancer therapy.

## 1. Introduction

The discovery of the carcinoembryonic antigen (CEA) as a tumor marker for colorectal carcinoma in 1965 by Gold and Freedman [1] was the milestone for identifying a much wider family of 12 carcinoembryonic antigen-related cell adhesion molecules (CEACAMs) which mediate intricate mechanisms of modulation and dysregulation during complex biological processes regarding cancer progression, inflammation, metastasis, and angiogenesis [2].

The most vastly distributed protein within this family is CEACAM1, being expressed on various normal epithelia from the gastrointestinal tract (*intestinal and colonic superficial epithelial cells, duodenal Brunner glands, esophageal glands, bile, and pancreatic ducts*), prostatic glands, gall bladder, mammary ducts, endometrium, renal tubuli, extravillous trophoblast, etc., as well as endothelial cells, natural killer (NK) cells, T and B lymphocytes, and myeloid cells [3–9].

Subjected to alternative splicing, the CEACAM1 primary transcript generates 12 different human isoforms, 3 of which are secreted versions that play an important role in inhibition of intercellular adhesion, being a marker of melanoma, pancreatic, and urothelial bladder carcinoma (UCB) progression [10–12]. CEACAM1 alternative splicing also results in generation of two major cytoplasmic domains, the so-called long (-L) and short (-S) tails, both of which have dysregulated expression in colorectal (CRC), breast, and non-small-cell lung carcinomas (NSCLC) [13–15].

CEACAM1 splice variants differ with respect to the number of extracellular domains and type of intracellular cytoplasmic domains [16]. The extracellular domains consist of one amino-terminal immunoglobulin variable-region-like (IgV-like) domain, which mediates hemophilic or heterophilic interactions [17, 18], and a maximum of three immunoglobulin constant-region-type-2-like (IgC2-like) domains, whose roles are still unclear. Regarding the intracellular cytoplasmic domains, splicing connects the various isoforms to either a long cytoplasmic tail (-L) or a short cytoplasmic tail (-S) [16]. (-L) tails contain two immunoreceptor tyrosine-based inhibitory motifs (ITIMs) that coordinate inhibitory signaling via Src homology 2 domain-containing tyrosine phosphatase- (SHP-) 1 or (SHP-) 2 recruitment following phosphorylation by Src family tyrosine kinases [19]; (-S) tails lack ITIMs [16].

SHP-1 and SHP-2 act as nonreceptor tyrosine phosphatases, which reverse critical tyrosine phosphorylation reactions induced by the action of tyrosine kinases [20], thus promoting signal inhibition. As a consequence, ITIM-containing family members of CEACAM1 (CEACAM1-L) generally mediate negative signaling, while ITIM-deficient CEACAM1 (CEACAM1-S) isoforms do not [5]. An increase of the CEACAM1-L/CEACAM1-S ratio is associated with decreased proliferation of tumor cells [21].

Isoform expression of CEACAM1 in tumoral tissue is particularly dynamic showing considerable downregulation within the epithelia in the early phases of many solid cancers such as prostate [22], colon [23, 24], breast [25], and liver [26] carcinomas. Restoration of CEACAM1 expression in tumor cell lines often abolishes their oncogenicity *in vivo* as indicated by studies in syngeneic or immune-deficient mice, where reinsertion of various CEACAM1 isoforms in colorectal or prostate CEACAM1-negative tumor cells proved CEACAM1-L expression to be essential for maintaining a normal phenotype with the inhibition of allograft or xenograft tumor development [27, 28]. Therefore, this adhesion molecule has been regarded as a tumor suppressor.

In contrast, studies showing CEACAM1-L overexpression in advanced stages of malignancies such as melanoma [29], NSCLC [30], bladder [31], CRC [32, 33], thyroid [34], and gastric [35] carcinomas correlate high abundance of CEACAM1-L with potential of invasiveness and metastatic spread [36], thus challenging the previously postulated concept of a tumor suppressive effect of this adhesion molecule.

This discrepancy is partially explained by the fact that ITIMs also bind Src family kinases (SFKs), which play critical signaling roles in hematopoietic cell function, such as B cells, T cells, NK cells, monocytes, granulocytes, and mast cell activation [37]. CEACAM1-SFK interactions contribute to cell adhesion properties of eosinophils as well as tumor cells [38–40]. Studies assessing CEACAM1 isoform expression in human neoplastic mast cells (mastocytosis) and medullary thyroid carcinoma cell (MTC) lines suggest that CEACAM1-L enhances cell growth in association with preferential interactions and activation of SFKs [16]. Thus, the dominantly interacting proteins SHP1 or SFK determine whether CEACAM1-L displays a positive or negative role in tumor cells [16].

## 2. The Role of CEACAM1 in the Regulation of Immune Surveillance, Immune Evasion, and Inflammation

The decreased expression of CEACAM1 in early malignancies and its upregulation in advanced cancers were difficult to reconcile. Further arguments supporting the molecule's role in tumor growth and progression are revealed by closer insights into the relationship of CEACAM1 with cells of the immune compartment, particularly T and NK cells, which illustrate its capacity to evade the immune system.

CEACAM1 homophilic interactions and the CEACAM1 heterophilic interactions with CEACAM5 inhibit NK-mediated killing independently of major histocompatibility complex (MHC) class I recognition and also interfere with the interferon-*γ* (IFN-*γ*) release activities of NK cells, as well as tumor-infiltrating lymphocytes (TILs) [6, 41–43]. These findings are supported by mouse cancer models, where CEACAM1 silencing results in upregulated NK cell activating ligands on the cancer cell surface [44]. In mouse melanoma cell lines, CEACAM1-4L isoform downregulates cell surface levels of NKG2D ligands MICA and ULBP2 [45] while CEACAM1-3S and -3L overexpressions in CRC cell lines cause sequestration of MICA/B intracellularly, preventing it from activating NK cells [44].

The essential interplay between CEACAM1 and cells of the immune system is well documented in melanoma. High percentages of circulating NK and CD8+ lymphocytes are CEACAM1+ in melanoma patients [10]. CEACAM1 homophilic interactions between TILs and melanoma cells appear to dampen *in vivo* TIL functions, limiting the efficacy of TIL adoptive cell transfer therapy in melanoma patients [46]. A study model investigating aspects of melanoma cell long coincubation with antigen-specific TIL demonstrated that the surviving melanoma cells increase their surface CEACAM1 expression, which in turn confers enhanced resistance against fresh TIL [46]. This appears to be an active process, driven by specific immune recognition, and is at least partially mediated by lymphocyte-derived IFN-*γ* [46]. These findings are consistent with results from CRC studies where CEACAM1 expression on TILs in mice and humans marks the most highly exhausted T cells [47]. Circulating CD8+ T lymphocytes and TILs from CRC patients have overexpression of CEACAM1 and TIM-3 compared with normal tissue [47]. CRC tumors among the double-positive (CEACAM1^+^TIM-3^+^) T cells exhibit a significant decrease in IFN-*γ* production [47]. These well-defined inhibitory roles of CEACAM1 in T and NK cells present the molecule as a valuable target for cancer immunotherapy with monoclonal antibodies in late-stage cancer.

CEACAM1's crucial role in regulating autoimmunity and antitumor immunity is also portrayed by analyzing its interaction with T cell immunoglobulin domain and mucin domain-3 (TIM-3), which endows TIM-3 with T cell inhibition capacity [18]. TIM-3 is an activation-induced inhibitory molecule involved in tolerance shown to induce T cell exhaustion in chronic viral infection and cancers [48–52], which under some conditions may also be stimulatory. Mouse adoptive transfer colitis models show CEACAM1-deficient T cells to be hyperinflammatory with reduced cell surface expression of TIM-3 as well as regulatory cytokines, and this can be restored by T cell-specific CEACAM1 expression [53]. By forming heterodimeric interaction in both *cis* and *trans* through their (IgV)-like N-terminal domains, CEACAM1 facilitates the maturation and cell surface expression of TIM-3, resulting in its tolerance-inducing function [18]. CRC cancer mouse models demonstrate that coblockade of CEACAM1 and TIM-3 leads to enhancement of antitumor immune responses and improved elimination of tumors [18].

The involvement of CEACAM1 in angiogenesis is also important for its protumorigenic effect. CEACAM1 was shown to be a major effector of vascular endothelial growth factor (VEGF) in early tumor microvessel formation [54]. Kilic and collaborators demonstrated that VEGF increases CEACAM1 expression on both mRNA and protein levels, and the administration of a monoclonal CEACAM1 antibody blocks *in vitro* VEGF-induced endothelial tube formation [54, 55]. Furthermore, by using an experimental mouse model of cutaneous leishmaniasis, a disease known to produce severe local inflammation accompanied by accumulation of CD11b cells at the site of infection, a VEGF-independent role of CEACAM1 has been characterized [56]. Both blood and lymphatic vessel formation appear to be affected by the loss of CEACAM1/CD11b cells, which control angiogenesis during inflammation [56]. Due to its capacity to evade the immune system, as well as its potent proangiogenic effects, CEACAM1 appears to play an important role in tumor growth and progression.

The emerging picture of CEACAM1 is extremely complex as its role in tumor cells appears to be contradictory, supporting both down- and upregulation. Detailed investigation of the expression patterns of this adhesion molecule in different cancer types is crucial in determining CEACAM1 involvement in carcinogenesis and possible significance of its altered expression in terms of diagnosis, prognosis, and treatment of distinct human neoplasms.

Our review focuses on emphasizing recent insights into the role of CEACAM1 in various cancer types due to their importance in designing a more comprehensive role in cell transformation of this adhesion molecule which may be a feasible target potentially leading to promising strategies in cancer treatment.

## 3. Malignancies Showing Early Phase CEACAM1 Downregulation

### 3.1. Colorectal Carcinoma

CEACAM1-reduced expression has been reported in more than 85% of early colorectal adenomas and carcinomas [57, 58] leading to a supposed tumor growth inhibitor function of this adhesion molecule in CRC development. Hyperplastic polyp lesions and aberrant crypt foci, the earliest stages of CRC, also have reduced levels of expression of CEACAM1 [59]. Experimental murine models show similar results, with CEACAM1 knockout mice developing a significantly greater number of colonic tumors than their controls when treated with azoxymethane to induce tumorigenesis [60].

However, studies using specific anti-CEACAM1 antibodies revealed elevated expression in high-grade adenomas, adenocarcinomas, and metastatic CRC [32, 33] suggesting a bimodal role in CRC progression. In addition, Ieda et al. highlighted the CEACAM1-L isoform presence as an independent risk factor for CRC hematogenous and lymph node metastasis and for a short survival time [61].

### 3.2. Mammary Gland Carcinoma

While expressed prominently on the luminal side of healthy mammary epithelia, CEACAM1 becomes randomly distributed on the cell surface or is completely lost during mammary cancer progression when typical architectural features of polarized tissues decline [62]. Similar to previous observations made with colorectal cancer, a low CEACAM1 expression was found in about 65% cases among breast cancer tissues in comparison to adjacent normal breast tissue [63]. A study by Wang et al. shows cancer tissues from 60 patients with mammary carcinoma to exhibit no CEACAM1 staining (12/60 patients 20%) or weak CEACAM1 expression (13/60 patients 21.7%) while the adjacent breast tissues show moderate to intense staining in most cases, without negative expression [63].

On the other hand, a study by Gerstel and collaborators comparing the intratumoral vascular tree in spontaneous and transplanted mammary adenocarcinomas in CEACAM1 competent mice with CEACAM1 null hosts shows the former to have increased vascular densities and pericyte coverage, with increased tumor vascularization and angiogenesis [64]. CEACAM1 was only expressed in peritumoral vessels [64]. The authors conclude that endothelial expression of CEACAM1 in the vasculature of the mammary tumor periphery appears to be an important component building a proangiogenic microenvironment that supports tumor vessel stabilization [64].

### 3.3. Bladder and Prostate Carcinomas

Immunohistochemical studies conducted by Oliveira-Ferrer and coworkers [31] in 2004 on nonmalignant urinary bladder tissues revealed “umbrella cells”-epithelial cells lining the luminal surface of transitional epithelium to exhibit CEACAM1 staining, while blood vessels of the normal bladder appeared negative. On the contrary, in early tumor stage pTa, CEACAM1 immunostaining became negative in tumoral cells, whereas the majority of blood vessels closely associated with or growing into the epithelial layer containing tumor cells were found CEACAM1-positive. As for invasive bladder tumors, all cases showed CEACAM1-positive blood vessels in close association with the tumor cell groups, with few tumoral cells and neighboring normal urothelial area still exhibiting CEACAM1 expression. Thus, CEACAM1 expression appears to be downregulated in bladder cancer cells, while concurrently upregulated in endothelial cells of tumoral adjacent blood vessels.

Tilki and collaborators made similar observations in prostate cancer, conducting electron microscope studies on prostate intraepithelial neoplasia (PIN) specimens [65]. CEACAM1 expression was found to be downregulated in epithelial dysplastic cells and upregulated in adjacent endothelial cells, with concurrent VEGF-A, -C, and -D upregulation, indicating activation of angiogenesis.

Studies from Ergün and coworkers support the previous observations that the differential switch in CEACAM1 expression stimulates VEGF and fibroblast growth factor-dependent proangiogenic activities such as neocapillary formation, proliferation, and chemotaxis, thus, promoting angiogenesis [54].

Interestingly, CEACAM1 upregulation during endothelial cell angiogenic activation can be detected in both membrane-bound forms and supernatant of endothelial cells, suggesting that during this process, soluble CEACAM1 forms might be released into body fluids [54].

Based on this finding, another study conducted by Tilki and collaborators [12] attempted to assess whether CEACAM1 urinary levels could help differentiate patients with UCB from healthy subjects. After performing western blot analysis on urinary samples from 135 subjects (93 with UCB and 42 with no UCB), they concluded that urinary bladder carcinoma and an invasive stage are associated with higher urinary levels of CEACAM1.

The data presented displays the disappearance of CEACAM1 in the dysplastic epithelium as one of the earliest signs of tumors switching from superficial noninvasive and nonvascularized to invasive and vascularized [31]. This supports the previously postulated hypothesis that the CEACAM1 presence in normal epithelia functions as a tumor suppressor [27, 66, 67]. However, epithelial CEACAM1 downregulation increases the expression of proangiogenic factors [54, 55] and favors endothelial CEACAM1 upregulation [31], promoting invasiveness, hence the dual role of CEACAM1 expression in these malignancies.

## 4. Malignancies with CEACAM1 Overexpression

### 4.1. Non-Small-Cell Lung Carcinoma

Immunohistochemical and serum assessments of CEACAM1 in NSCLC revealed it is a valuable prognostic biomarker [68]. CEACAM1-S isoform and the CEACAM1S/CEACAM1-L ratio appear to be significantly higher in tumors than in normal tissue [68].

Sienel and collaborators found significant association between CEACAM1 expression and tumoral status, while trying to elucidate the role of CEACAM1 in the progression and survival of patients with operable NSCLC [69]. They analyzed the immunohistochemical expression of CEACAM1 in tumor samples from 145 patients with completely resected NSCLC. All sections of normal bronchiolar epithelium stained negative, 73 tumors (50.4%) displayed between 1 and 66% CEACAM1-positive tumoral cells, and the remaining 72 tumors (49.6%) showed even greater percentage of positive cells. Following a detailed statistical analysis, the authors concluded that the absence of CEACAM1 in normal lung tissue and its expression in tumor cells argue against a tumor suppressive role of CEACAM1 in NSCLC.

Furthermore, elevated CEACAM1 expression correlates with severe disease and tendency to reduced cancer-related survival of the total population, rather indicating CEACAM1 as a promoter of lung cancer progression [69]. In addition, urinary levels of CEACAM1 represent an excellent biomarker for NSCLC patients when considered alongside other signature proteins [70].

### 4.2. Pancreatic Adenocarcinoma

Various studies indicate CEACAM1 to be a useful biomarker for pancreatic adenocarcinomas (PAC), being present in both tumor specimens and serum of patients compared to healthy individuals [11, 71–73]. CEACAM1 is more sensitive and specific than the already consecrated CA 19-9 in differentiating cancer from normal controls, and this is improved by combining CEACAM1 and CA 19-9 [11].

Furthermore, CEACAM1 appears to be present early in the development of the disease, with most pancreatic intraductal neoplasia 3 (PanIN-3) lesions, representing pancreatic carcinoma in situ, showing elevated expression by immunohistochemical analysis [11]. As PACs have high lethality associated due to appearance of clinical manifestations late in the natural course of the disease [74], development of blood-based biomarkers capable of detecting PAC at early, preclinical stages is a necessity. A study conducted by Nolen et al. indicates CEACAM1 and prolactin as the earliest serum biomarkers to be detected at significantly altered levels, up to 35 months prior to diagnosis [75].

CEACAM1 has also been strongly correlated with distant metastasis of PAC [73].

### 4.3. Thyroid Carcinoma

CEACAM1 is not appreciably expressed in normal human thyroid tissue and is rarely present in benign tumors [4] but highly upregulated in thyroid carcinomas, especially in metastatic tumors [34]. To investigate the role of CEACAM1 in thyroid carcinomas, Liu and coworkers [34] compared the effects of this adhesion molecule in WRO cells (usually devoid of CEACAM1 expression) and clones with forced CEACAM1 expression. In vitro analysis revealed that forced expression of CEACAM1 caused a significant G0/G1 phase arrest, with enhanced cell–matrix adhesion and increased cell invasion, resulting in diminished tumor growth and increased tumor invasiveness when applied to a xenograft mouse model. Conversely, CEACAM1 downregulation stimulated MRO cell tumor growth with reduced invasiveness. They concluded CEACAM1 is an important cell proliferation inhibitor, retarding several parameters of tumoral growth while mediating invasion.

These findings are consistent with CEACAM1 expression correlation to invasiveness in cutaneous malignant melanoma [29] and to the molecule's implication in mediating trophoblast/endometrial interactions during trophoblastic invasion of the endometrium [76, 77].

### 4.4. Melanoma

Normally, melanocytes do not exhibit CEACAM1 [78, 79]. Concerning CEACAM1 expression in melanocytic nevi, few data is available. While assessing CEA family expression in melanocytic nevi using monoclonal and polyclonal panels of antibodies (none of which directed only towards CEACAM1), Egawa et al. demonstrated an increased expression of this protein family on the surface of both acquired and congenital melanocytic nevi, in a similar distribution pattern [79]. Interestingly, blue nevi, which are melanocytes from the neural crest that failed reaching the epidermis during embryological migration, do not stain positive for CEA [79].

Immunohistochemical studies conducted by Gambichler and collaborators comparing CEACAM1 expression in benign and malignant melanocytic tumors and normal peritumoral skin reported a progressive increase of median CEACAM1 expression from 1% in benign nevi and 9.6% in dysplastic nevi to 18% in thin superficial melanomas (defined as melanomas with Breslow tumor thickness <1 mm) and 74% in thick superficial spreading melanomas (defined as melanomas with Breslow tumor thickness >1 mm) (*p* < 0.0001) [80]. Peritumoral melanocytes from normal skin stained negative [80].

In melanoma, the role of CEACAM1 is well established, with a significant number of studies unanimously demonstrating its prognostic value in diagnosis, progression, and metastasis [29, 80, 81]. There is an overwhelming expression of the CEACAM1-L isoform on melanoma cells [45, 82], and elevated serum CEACAM1 levels were found to positively correlate with decreased patient's survival, failure to respond to immunotherapy, and decreased efficacy of autologous vaccination [10, 83, 84].

In metastatic cutaneous melanoma, 89% of lesions express CEACAM1, and CEACAM1 expression increases during tumor progression [82], while soluble CEACAM1 levels significantly correlate with the level of LDH [10].

The overwhelming evidence of CEACAM1 importance in melanoma solidify the claim that this adhesion molecule could be applied as an improved prognostic and predictive biomarker for melanoma patients over the commonly used Breslow depth [85].

### 4.5. Squamous Cell Carcinoma (SCC)

There is a lack of data concerning CEACAM1 expression in keratinocytic tumors. A study by Wang and collaborators demonstrated that CEACAM1 overexpression and abundance of neutrophils could predict a poor clinical outcome in tongue squamous cell carcinoma (TSCC) patients [86]. In this study, CEACAM1 expression on tumor cells and increased neutrophils infiltration were associated. The proposed mechanism of CEACAM1 overexpression attracting more neutrophils to tumor sites is through IL-8 and CXCL-6 upregulation. This finding is consistent with results from other studies that link cytokine-induced CEACAM1 expression on keratinocytes to a prolonged lifespan of neutrophils [87].

However, the role of CEACAM1 appears to be dependent of its distribution in oral squamous cell carcinoma. Zhou et al. showed membranous CEACAM1 expression inhibits angiogenesis and lymphangiogenesis and is associated with well-differentiated SCC [88]. Conversely, cytoplasmic CEACAM1 expression, which is associated with poorly and moderately differentiated SCCs, promotes angiogenesis and lymphangiogenesis by mediating the transformation of vascular endothelial cells into lymphatic endothelial cells.

## 5. Conclusions

The emerging picture of CEACAM1 in the context of cancer and the immune system is very complex. Even though decades of studies have tried to characterize its role in carcinogenesis, there is no general agreement upon this adhesion molecule's behaviour in human tumors. CEACAM1 works together with specific signaling factors, proteins and receptors, dependent on the various contexts in which it occurs; therefore, its role should be interpreted separately in each of these different cell types, tissues, and pathological conditions. CEACAM1 appears to be a valuable prognostic factor in various tumors through its different expression patterns on cancer cells. In addition, its roles in tumor progression, immune evasion, angiogenesis, and invasion make it a promising molecular target that can be exploited alongside other existing immunotherapeutics as specific cancer therapies.
